# A Stapled Peptide Mimic of the Pseudosubstrate Inhibitor PKI Inhibits Protein Kinase A

**DOI:** 10.3390/molecules24081567

**Published:** 2019-04-20

**Authors:** Jascha T. Manschwetus, George N. Bendzunas, Ameya J. Limaye, Matthias J. Knape, Friedrich W. Herberg, Eileen J. Kennedy

**Affiliations:** 1 Department of Biochemistry, Institute for Biology, University of Kassel, Heinrich-Plett-Str. 40, 34132 Kassel, Germany; j.manschwetus@uni-kassel.de (J.T.M.); maknape@googlemail.com (M.J.K.); 2 Department of Pharmaceutical and Biomedical Sciences, College of Pharmacy, University of Georgia, 240 W. Green St, Athens, GA 30602, USA; georgenb@uga.edu (G.N.B.); ameya.limaye@uga.edu (A.J.L.)

**Keywords:** PKA, stapled peptide, PKI, pseudosubstrate, kinase inhibitor, IP20

## Abstract

Kinases regulate multiple and diverse signaling pathways and misregulation is implicated in a multitude of diseases. Although significant efforts have been put forth to develop kinase-specific inhibitors, specificity remains a challenge. As an alternative to catalytic inhibition, allosteric inhibitors can target areas on the surface of an enzyme, thereby providing additional target diversity. Using cAMP-dependent protein kinase A (PKA) as a model system, we sought to develop a hydrocarbon-stapled peptide targeting the pseudosubstrate domain of the kinase. A library of peptides was designed from a Protein Kinase Inhibitor (PKI), a naturally encoded protein that serves as a pseudosubstrate inhibitor for PKA. The binding properties of these peptide analogs were characterized by fluorescence polarization and surface plasmon resonance, and two compounds were identified with K_D_ values in the 500–600 pM range. In kinase activity assays, both compounds demonstrated inhibition with 25–35 nM IC_50_ values. They were also found to permeate cells and localize within the cytoplasm and inhibited PKA activity within the cellular environment. To the best of our knowledge, these stapled peptide inhibitors represent some of the highest affinity binders reported to date for hydrocarbon stapled peptides.

## 1. Introduction

Protein kinases play pivotal roles as key modulators of cellular signaling events and are involved in numerous and diverse processes, including hormone response signaling, gene transcription, cell differentiation and apoptosis [1]. More than 500 eukaryotic protein kinases are encoded by the human genome and thus tight regulation of enzymatic activity and substrate interactions is essential for proper kinase function [2]. Aberrant signaling by this important enzyme class is linked to a broad spectrum of health issues such as cancer, diabetes and neurodegenerative diseases [3]. Due to the wide-ranging implications by kinases in signaling and biology, significant efforts have been put forth to develop selective kinase inhibitors that can be applied as either research tools or therapeutic inhibitors [4]. The majority of kinase inhibitors target the ATP binding site as it forms a deep pocket that is amenable for small molecule targeting [3]. Since the ATP pocket resides within the kinase domain and is therefore conserved across the kinase superfamily [5], specificity has remained a challenge. Alternatively, allosteric inhibitors may overcome the challenge of specificity by targeting more evolutionarily divergent surfaces or pockets for a kinase of interest.

As a model system, we focused on one of the best understood kinases, cAMP-dependent protein kinase (PKA). The PKA tetrameric holoenzyme complex consists of a regulatory subunit dimer and two monomeric catalytic subunits [6]. The catalytically inactive holoenzyme is activated by increased levels of the second messenger cAMP upon extracellular or intracellular stimuli, triggering the R subunits to undergo a conformational change that can then result in release of the catalytic subunits (PKA-C) [7]. PKA-C phosphorylates a vast variety of intracellular substrates that regulate a myriad of cellular processes via phosphorylation of the consensus sequence Arg-Arg-X-Ser/Thr-y, where X represents a small residue and y represents a large hydrophobic residue [8]. 

**Figure 1:**
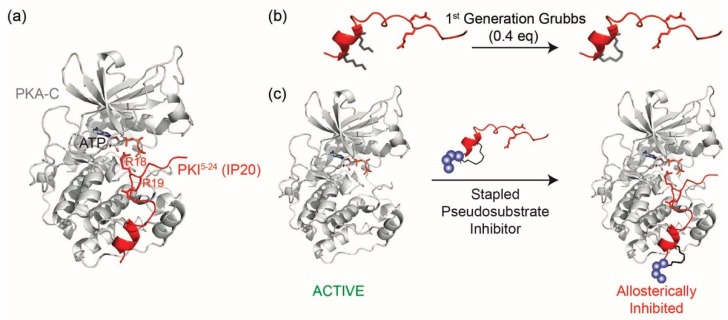
Design of a stapled pseudosubstrate inhibitor for the catalytic subunit of Protein Kinase A (PKA-C): (**a**) Crystal structure of PKA-C (gray, PDB ID: 1ATP) bound to a Protein Kinase Inhibitor (PKI^5–24^ ) (IP20, red). The side chains of Arg 18 and 19 are shown and are critical for pseudosubstrate inhibition; (**b**) Peptide stapling was performed on-resin with ring-closing metathesis chemistry using the 1st Generation Grubbs catalyst to introduce a staple in the N-terminal alpha-helix; and (**c**) An analog of IP20 is designed by incorporating a hydrocarbon staple into the N-terminus of the PKI-derived peptide to serve as a non-catalytic, allosteric inhibitor for PKA-C. Structures were rendered using PyMol.

Nearly 50 years ago, a naturally encoded protein was discovered that could inhibit the catalytic activity of PKA, termed Protein Kinase Inhibitor (PKI) [9]. It was later discovered that only a short fragment of PKI was required for inhibition [10,11,12,13]. This 20-residue fragment derived from the N-terminal residues 5–24 of PKI, termed IP20, was found to be highly specific for PKA and could inhibit activity with K_D_ values in the single nanomolar range by binding the catalytic subunit of PKA as a pseudosubstrate, thereby preventing substrate engagement with the kinase (Figure 1a). IP20 includes a cluster of basic arginine residues as well as a hydrophobic portion in the pseudosubstrate sequence (Arg^15^-Thr-Gly-Arg-Arg-Asn-Ala-Ile^22^, where Ala substitutes for the phosphorylatable Ser/Thr). The charged residues, in particular the P-3 Arg, are a requisite for high-affinity binding to PKA-C [10,13,14] in addition to Mg^2+^ and ATP [14]. Although this 20-residue peptide, IP20 (PKI^5–24^), has been used for many years as an investigative tool in biochemical assays, the peptide itself is not membrane permeable and requires modifications such as myristoylation to promote cell permeation [15,16]. However, a major drawback of this reagent is that the hydrophobic nature of the myristoylation moiety can intrinsically promote membrane interactions/embedding, thereby leading to potential mislocalization of the peptide and limiting its interactions with PKA-C at various intracellular locations.

Due to its high target specificity and affinity for PKA-C, we explored whether the PKA pseudosubstrate peptide IP20 could be modified through hydrocarbon peptide stapling to improve cell permeability in the absence of lipidation (Figure 1b,c). Several analogs were designed and characterized for their affinity to PKA-C. While the stapled analogs of IP20 were found to have worsened affinities for PKA-C, we found that elongation of the sequence and reposition of the staple restored affinities to mid-picomolar range as measured by both fluorescence polarization (FP) and Surface Plasmon Resonance (SPR), representing one of the highest affinity binders reported to date for hydrocarbon stapled peptides. These peptides were further shown to inhibit the catalytic activity of PKA-C in vitro and could permeate cells and inhibit PKA phosphorylation within the cellular environment. Thus, the constrained peptides developed in this study represent a novel, non-lipidated, cell permeable tool for allosteric inhibition of PKA.

## 2. Results and Discussion

In order to determine whether a constrained peptide could be developed to mimic the pseudosubstrate inhibitory properties of PKI, analogs of the 20-mer peptide IP20 were first designed (compounds **1**–**4**, Table 1). Based on the crystal structure of IP20 with PKA-C, the peptide largely interacts with the catalytic subunit in an elongated fashion that lacks an ordered secondary structure [17]. However, a single alpha-helical turn is present in the N-terminus of the peptide structure (PKI residues 5–13) and thus provided a potential point for incorporation of the hydrocarbon staple. Based on this, an olefinic amino acid ((*S*)-*N*-Fmoc-2-(4′-pentenyl) alanine) was introduced into positions four and eight of the 20 amino acid peptide sequence so as to constrain a portion of the peptide while trying to minimize any structural impact on the C-terminal portion of the sequence. Peptides were synthesized using standard Fmoc solid phase chemistry. The olefinic amino acids were cyclized on solid support using ring closing metathesis (RCM) chemistry with 0.4 equivalents of 1st Generation Grubbs catalyst for two 1-h treatments in 1,2-dichloroethane (DCE) (Figure 1b). Cyclization yields ranged from 87–98% for each of the peptide products. Additionally, the N-terminus was modified to contain either a hydrophobic linker (β-alanine, βA, compound **2**) or hydrophilic linker (PEG_3_, compound 4). Non-stapled peptides bearing the same N-terminal modifications were also synthesized as controls (compounds 1 and 3). Peptides products were confirmed by ESI-MS and purified by RP-HPLC over a Zorbax SB-C18 column prior to use. Overall yields ranged from 1–2%.

**Figure 2:**
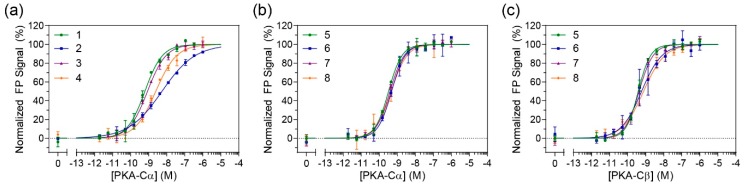
Direct binding measurements of fluorescein-labeled peptides by fluorescence polarization (FP): (**a**) Binding measurements of PKI^5-24^ analogs to PKA-Cα demonstrate that stapling the 20-residue peptide negatively impacts binding; (**b**) Binding measurements of PKI^1–24^ analogs to PKA-Cα show no negative effect when stapling the 24-residue peptide; and (**c**) Binding measurements of PKI^1–24^ analogs to PKA-Cβ1 reveal that the stapled 24-mer analogs also retain high affinity for the Cβ1 isoform.

In order to determine whether the constrained peptides retained their binding affinity towards PKA-C, FP studies were performed (Figure 2a and Figure S5). N-terminally fluorescein-labeled peptides were incubated with a concentration range of recombinant human PKA-Cα. The non-stapled controls (compounds **1** and **3**) had measured K_D_ values ranging from 0.7–0.8 nM which is comparable to the previously reported K_D_ value of 1 nM by IP20 for PKA-Cα [18]. Unfortunately, the stapled versions of IP20 (compounds **2** and **4**) appeared to detrimentally affect binding for PKA-Cα with K_D_ values ranging around 5–5.5 nM (Table 1). A one-way ANOVA (Tukey test) showed that the approximately 7-fold decrease in affinity was significant.

Based on these findings, we sought to explore whether the parent IP20 peptide could be modified in such a way that the staple wouldn’t detrimentally affect its binding towards PKA-C. It was previously shown that extending IP20 by four residues (PKI^1–24^) had inhibitory properties on PKA-C that were on par with IP20 [12], however, this would provide additional space to shift the staple closer to the N-terminus. Using PKI^1–24^ as a parent sequence, a subsequent set of stapled analogs was synthesized as before with either a βA or PEG_3_ linker on the N-terminus (compounds 6 and 8). Non-stapled analogous controls were also synthesized (compounds **5** and **7**). FP measurements were subsequently performed on FAM-labeled peptides with PKA-Cα (Figure 2b). Under these conditions, no notable differences could be detected between the stapled and unstapled versions, indicating that the introduction of a staple to this longer sequence did not impede binding to PKA-C. Further, as compared to the PKI^5–24^ analogs, binding affinities were notably improved with K_D_ values ranging from 600–700 pM. To determine whether these stapled compounds could detect other PKA-C isoforms, they were also tested for binding to PKA-Cβ1 (Figure 2c and Table 1). Under these conditions, there was no significant loss in affinities as compared to their non-stapled counterparts, and all peptides retained K_D_ values in the mid-high picomolar range. 

**Figure 3:**
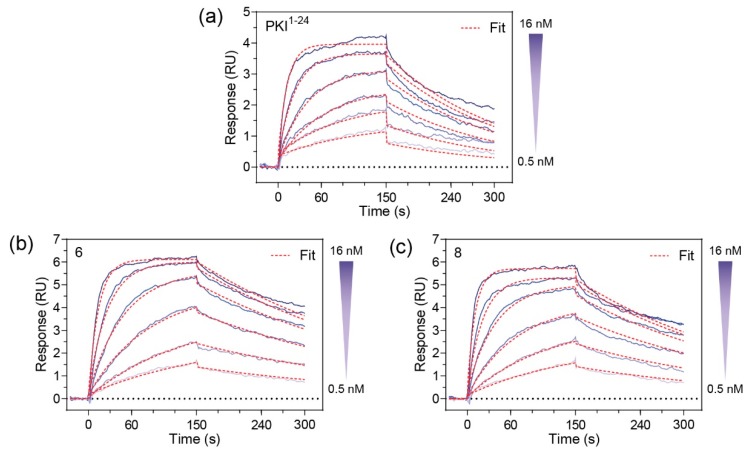
Binding interaction studies by Surface Plasmon Resonance (SPR): (**a**) Binding measurements of the non-modified PKI^1–24^ control; (**b**) Binding measurements of Compound **6**; and (**c**) Binding measurements of Compound **8**. Compounds **6** and **8** demonstrated K_D_ values of 500 and 600 pM, respectively and demonstrate slightly slower dissociation rates as compared to the non-modified PKI^1–24^ control.

Since **6** and **8** were found to have sub-nanomolar affinities for PKA-C, we wanted to kinetically characterize this interaction using SPR. For this, an N-terminally GST-tagged PKA-C (GST-PKA-Cα) was captured on a CM5 chip surface via immobilized α-GST antibodies. The test compounds **6** and **8** were injected as an analyte over a concentration ranging from 0.05 to 28 nM. Association and dissociation rate constants were determined using a 1:1 Langmuir binding model. Both compounds **6** and **8** were found to have K_D_ values in the 500–600 pM range (Figure 3). As compared to the unstapled parent control peptide (PKI^1–24^), both stapled analogs appear to have improved affinities for PKA-Cα. Notably, while the stapled and parent compounds were found to have similar association rates, it appears that the non-stapled peptide has significantly faster dissociation rates as identified using one-way ANOVA analysis (Dunnet test), resulting in an overall reduction in K_D_ (Table 2 and Table S1).

**Figure 4:**
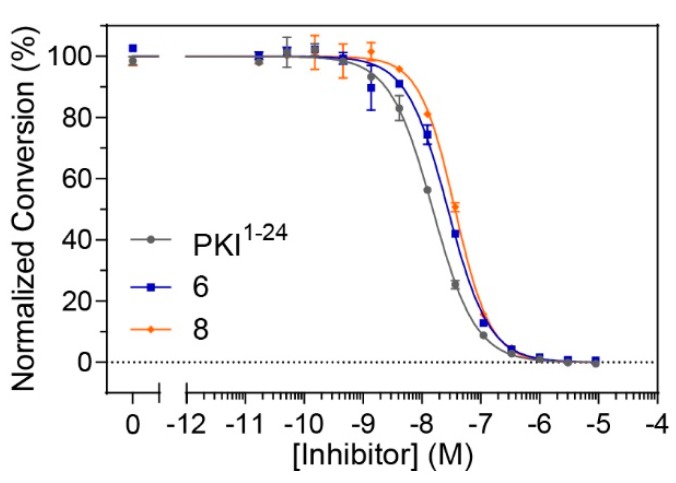
Kinase activity assays using microfluidic electrophoretic mobility shift assays (MMSA): Phosphorylation of a fluorescently-labeled substrate peptide by PKA-Cα was monitored over a concentration range of inhibitor peptides **6** and **8** as well as PKI^1–24^. Both **6** and **8** were found to have IC_50_ values that compare to the non-modified parent control, PKI^1–24^, thereby indicating that the introduction of a staple does not impair its inhibitory activity on PKA-Cα.

Although constrained peptides **6** and **8** demonstrated high affinities for their target, PKA-C, it was unclear whether they could effectively inhibit the catalytic activity of their target kinase. In order to assess whether these constrained peptides could still serve as pseudosubstrate inhibitors, microfluidic electrophoretic mobility shift assays (MMSA) were used to analyze kinase function by monitoring phosphorylation of a substrate peptide (Kemptide, LRRASLG) using recombinant PKA-Cα (Figure 4 and Figure S7). Peptides **6** and **8**, along with the non-constrained peptide control (PKI^1–24^) were applied in serial dilutions ranging from 17 pM to 9 μM to determine half-maximal inhibitory concentrations (IC_50_ values). While the non-modified control was found to have an IC_50_ value of 17 nM, the stapled versions **6** and **8** had values ranging from 25–35 nM (Table 3 and Table S2). Overall, it appears that the stapled peptides are comparable to the non-modified parent peptide under substrate saturation conditions and still have considerable potency for kinase inhibition in the lower nanomolar range. 

**Figure 5:**
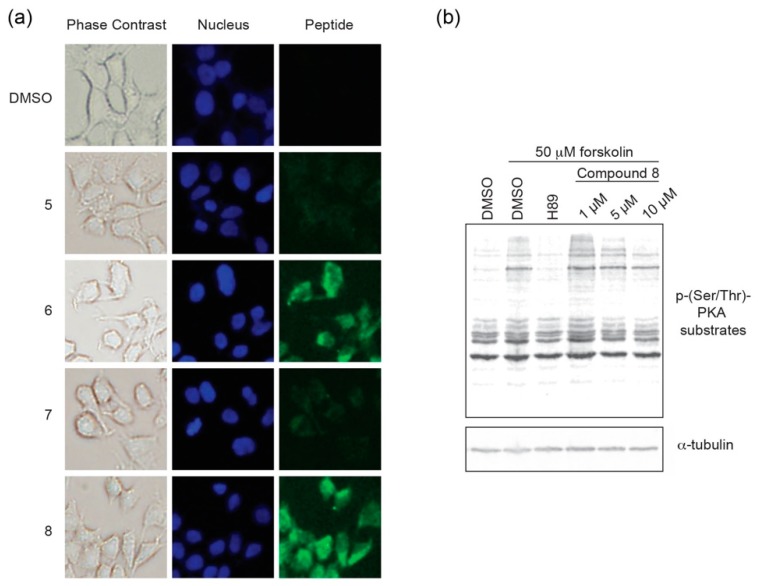
Cell-based uptake and inhibition: (**a**) Cell permeation is detected for stapled compounds **6** and **8** but not their unstapled counterparts after an 8 h incubation period; and (**b**) Cell-based inhibition by monitoring PKA activity in cells. In the presence of **8**, PKA substrate phosphorylation is inhibited in a dose-dependent manner.

Following in vitro characterization of these peptides, we next wanted to determine whether the staple would provide additional benefits for cell-based assays including improved cell permeation and *in cellulo* inhibition. Cell permeation experiments were performed using HEK293 cells. Cells were grown on chamber slides in complete media and 5 μM of each respective peptide analog of PKI^1–24^ was added to the media. Following an 8 h incubation, cells were imaged to monitor for intracellular localization (Figure 5a, Figures S8 and S9). While the stapled versions **6** and **8** were found to readily permeate cells, their non-stapled counterparts (**5** and **7**) were not notably detected in cells. 

Based on the cell uptake experiments, coupled with the observation that **8** appeared to have greater solubility in aqueous cell-based assays, we chose to further characterize **8** in a cell-based inhibition assay (Figure 5b). Following an 18 h incubation period in serum-free media to downregulate intrinsic PKA activity, HEK293 cells were pre-treated with compound **8** at different concentrations for 6 h. At this 24 h time point, cells were stimulated with forskolin, an adenylyl cyclase activator, to stimulate PKA activity for 30 min prior to lysis. The ATP-competitive catalytic inhibitor H89 (50 μM) was used as a negative control. PKA activity was monitored as a function of substrate phosphorylation using a phospho-Ser/Thr-PKA substrate antibody and tubulin was detected as a loading control. In the absence of stimulation, PKA substrate phosphorylation is downregulated to a basal level that is comparable to forskolin-stimulated cells that are co-treated with H89. Constrained peptide **8** was found to inhibit PKA substrate phosphorylation in a dose-dependent manner with a notable decrease in phosphorylated substrates at the 5 and 10 μM dosing range. Taken together, it appears that compound **8** can act as a cell permeable pseudosubstrate inhibitor of PKA-C. 

Since protein kinases are key regulators of diverse signaling pathways and diseases, they are attractive targets for manipulation both in basic research as well as therapeutic intervention. Significant efforts have been put forth to develop inhibitors/modulators of kinase activity, however the majority of these compounds target the highly conserved ATP pocket and numerous shortcomings have been noted including lack of specificity and therefore cross-reactivity, poor inhibitory potency, and clinical usage often results in rapid development of resistance [5]. 

As a research tool, the ATP-competitive small molecule inhibitor H89 has been widely used as a PKA-C inhibitor due to its ability to readily permeate cells and its K_i_ of 48 nM [19]. However, H89 was found to not only inhibit PKA-C but was also shown to inhibit other kinases with even greater potency than PKA [20]. After short peptides derived from PKI were found to inhibit PKA-C with high specificity [12], they became valuable research tools for in vitro studies. A shortcoming of these peptides is that they are not intrinsically cell permeable, however a derivative was later developed that contained the addition of a myristoyl group (myr-PKI^14–22^) [16]. The addition of the myristoyl moiety to PKI-derived peptides may significantly alter its interactions within a cellular environment, and thus alternative analogs lacking this moiety would expand the repertoire of reagents available for studying PKA-C in cells. Furthermore, several other kinases also contain a pseudosubstrate domain analogous to PKA-C including PKC and PKG [16,21] and thus this domain may serve more broadly as a viable target for selective, allosteric kinase inhibition. An alternative strategy has been employed by generating bi-substrate inhibitors of PKA where an ATP-competitive small molecule is conjugated to a peptidic moiety [22]. While some adenosine-oligoarginine conjugates (ARCs) were found to have high potency with K_D_ values as low as 3 pM and IC_50_ values in the low nanomolar range due to avidity effects [23], ease of synthesis of these compounds and cell permeation remains a challenge. 

In contrast to ATP-competitive inhibitors, kinase-targeting peptide inhibitors often mimic protein-protein interaction sites [24,25,26]. Peptide-based inhibitors unite the benefits of both small-molecules and proteins, potentially resulting in high specificity, high potency, membrane permeability, conformational restriction and metabolic stability [27]. The compounds developed in this study offer many advantages for PKA targeting including ease of synthesis and its ability to permeate cells without the need for other modifications. Moreover, these peptides have an extremely high affinity for their target. The SPR data fit very well with a 1:1 model of Langmuir binding and the association rates detected for **6** and **8** approach the limitations of the Biacore instrumentation. The stapled peptide affinities are surprisingly comparable to the full-length PKI protein which has an affinity of approximately 0.5 nM as measured by SPR (Figure S6 and [28]). The stapled peptides developed in this work can be applied as unique tools for various investigations such as competitive displacement studies with substrates and pseudosubstrates. Additionally, one could envision taking advantage of its high affinity by further functionalizing it into various forms including a proteolysis-targeting chimera (PROTAC) or bi-substrate inhibitors.

## 3. Materials and Methods

### 3.1. General Information

Standard N-a-Fmoc amino acids and rink amide MBHA resin LL were purchased from Novabiochem (Millipore Sigma, Burlington, MA, USA). (*S*)-*N*-Fmoc-2-(4′-pentenyl) alanine (Fmoc-S_5_) and 1st Generation Grubbs catalyst (Bis(tricyclohexylphosphine)benzylidene ruthenium(IV) chloride) were purchased from Sigma. 2-(6-Chloro-1-*H*-benzotrizole-1-yl)-1,1,3,3-tetramethylaminium hexafluorophosphate (HCTU) and Fmoc-11-amino-3,6,9-trioxaundecanoic acid (Fmoc-PEG_3_) were purchased from ChemPep (Wellington, FL, USA). Unless noted, all other reagents and solvents were purchased from Fisher Scientific (Hampton, NH, USA), Carl Roth (Karsruhe, Germany), Sigma Aldrich (St. Louis, MO, USA), AppliChem (Darmstadt, Germany). and Merck (Darmstadt, Germany). HPLC-grade acetonitrile, trifluoroacetic acid and methanol were used for peptide purification by HPLC and MS analysis.

### 3.2. Cell Culture

HEK293 cells were cultured in Dulbecco’s Modified Eagle Medium (DMEM) with glucose and l-glutamine (Lonza, Alpharetta, GA, USA), 10% fetal bovine serum (Thermo Scientific, Waltham, MA, USA), and penicillin/streptomycin (VWR Life Science, Radnor, PA, USA).

### 3.3. Peptide Synthesis

Synthesis was performed on MBHA resin LL using standard solid phase synthesis. Deprotection steps were performed using 25% (*v*/*v*) piperidine in 1-methyl-2-pyrrolidinone (NMP) for 25 min with gentle agitation. Coupling reactions with standard amino acids were performed using 10 eq amino acid, 9.9 eq HCTU and 20 eq *N*,*N*-diisopropylethylamine (DIEA) at room temperature for at least 45 min. Couplings with Fmoc-S_5_ and Fmoc-PEG_3_ were performed using 4 eq at room temperature for at least 60 min. Ring-closing metathesis of the olefinic amino acids ((*S*)-*N*-Fmoc-2-(4′-pentenyl) alanine) was performed on-resin in 1,2-dichloroethane (DCE) with 0.4 eq of 1st Generation Grubbs catalyst for two 1 h treatments. For N-terminal 5(6)-caroboxyfluorescein (FAM) labeling, 2 eq FAM were added along with 1.8 eq HCTU and 4.6 eq DIEA in DMF overnight with gentle agitation. For N-terminal biotin labeling, 10 eq of D-Biotin (Anaspec) was added along with 9.9 eq HCTU and 20 eq DIEA in a 1:1 mixture of DMF and DMSO overnight. Resin cleavage was performed in 95% (*v*/*v*) trifluoroacetic acid (TFA), 2.5% (*v*/*v*) triisopropylsilane and 2.5% (*v*/*v*) water for 4–5 h at room temperature, followed by precipitation in methyl-*tert*-butyl ether at 4 °C.

Peptides were characterized by ESI-MS (Agilent 6120 Single Quadrupole, Santa Clara, CA, USA) after HPLC separation over a Zorbax analytical SB-C18 column (Agilent 1200). Peptides were separated by reversed phase HPLC over a 10–100% gradient of water:acetonitrile with 0.1% TFA using a linear gradient and flow rate of 0.5 mL/min. Purification was performed using the same conditions except over a semi-preparatory column with a flow rate of 4 ml/min. FAM-labeled peptides were quantified based on absorbance at 495 nm in 10 mM Tris pH 8 (Bio-Tek Synergy 2) using an extinction coefficient of e = 68,000 M^−1^cm^−1^ as previously described [29]. Biotin-labeled peptides were quantified by measuring decreased absorbance of the 2-hydroxyazobenzen-4′-carboxylic acid (HABA)-avidin complex at 500 nm. Spectra for compounds **5**–**8** are shown in Figures S1–S4.

Peptide masses for each FAM-labeled product are as follows: 1 = 2649.9 (expected mass = 2650.8); 2 = 2758.2 (expected mass = 2759.0); 3 = 2769.6 (expected mass = 2768.9); 4 = 2876.4 (expected mass = 2877.1); 5 = 3094.8 (expected mass = 3095.3); 6 = 3145.2 (expected mass = 3145.3); 7= 3212.8 (expected mass = 3213.4); and 8 = 3262.4 (expected mass = 3263.5). 

Peptide masses for each biotin-labeled product are as follows: 2 = 2625.9 (expected mass = 2627.0); 3 = 2635.8 (expected mass = 2636.9); 4 = 2744.4 (expected mass = 2745.1); 5 = 2962.2 (expected mass = 2963.2); 6 = 3012.6 (expected mass = 3013.4); 7 = 3080.4 (expected mass = 3081.4); and 8 = 3130.8 (expected mass = 3131.5).

Peptide mass for the unlabeled version of compound 5 unlabeled = 2665.2 (expected mass= 2665.8). Peptide mass for the PKI^5–24^ FF negative control (βA-TTY*DFI*SGRTGFFNAIHD): 2740.2 (expected mass = 2740.9).

### 3.4. Protein Expression and Preparation

Human wildtype PKA catalytic subunit, isoform α1 (PKA-Cα in pET30a), was expressed in *E. coli* T7 Express lysY/I^q^ cells, while both isoform β1 (PKA-Cβ in pET30a) and N-terminally GST-tagged murine isoform α1 (GST-PKA-Cα in pGEX-KG) were expressed in *E. coli* BL21-CodonPlus(DE3)-RIL cells for 16 h at RT after induction with 0.4 M IPTG. All PKA-C isoforms were purified using IP20-resin affinity chromatography as previously described [30]. All isoforms were stored in elution buffer (50 mM Tris-HCl pH 7.4, 200 mM l-arginine, 1 mM EDTA, 50 mM NaCl). Prior to further use, the buffer was exchanged to buffer A (20 mM MOPS pH 7, 150 mM NaCl, 2 mM β-mercaptoethanol) for both PKA-Cα and PKA-Cβ1. Full-length PKI was expressed and purified as previously described [31].

### 3.5. Fluorescence Polarization (FP)

Direct binding studies of peptides to PKA-C were performed using FP as previously described by Saldanha, et al. [32] and adapted by Bendzunas, et al. [33]. Each FAM-labeled peptide was plated to have a final concentration of 0.5 nM after the addition of protein in 384-well microtiter plates (BRAND GmbH + Co KG, BRANDplates, pureGrade, black). Three-fold dilution series of PKA-Cα or PKA-Cβ1, respectively, were mixed in the peptide wells in a 1:1 ratio to have at least 12 final concentrations ranging from 1 μM to 0.2 pM. The assay was performed in FP buffer (20 mM MOPS pH 7, 150 mM NaCl, 0.005 % CHAPS, 1 mM ATP, 10 mM MgCl_2_) at room temperature. Focus height (7.6 mm) and gain (35 mPol) adjustments were performed on reaction mixtures without PKA-C. The following settings were utilized on a CLARIOstar (BMG LABTECH) plate reader in a top optic format: excitation/emission/dichroic filters: 482–16 nm/530–40 nm/LP 504; 50 flashes per well; settling time: 0.1 s. GraphPad Prism 6.01 (GraphPad Software, San Diego, CA, USA) was used to perform non-linear regression (sigmoidal dose response) on FP signals which were plotted against log-scale protein concentrations to determine equilibrium dissociation constants (K_D_). At least two protein preparations were used for three independent duplicate measurements.

### 3.6. Surface Plasmon Resonance Spectroscopy (SPR)

GST-PKA-Cα was captured by polyclonal α-GST antibodies (Carl Roth) which were covalently immobilized on a Series S Sensor Chip CM5 (Biacore, GE Healthcare) surface using standard NHS/EDC chemistry with a Biacore T200 instrument (Biacore, GE Healthcare) to approximately 3000–13,000 response units (RU) as previously described [28]. Required dilutions of the analytes were injected to the flow cells containing GST-PKA-Cα before proceeding to a buffer or regeneration run, respectively. All measurements were simultaneously performed on reference flow-cells with α-GST Ab only and next to blank runs without analytes to be subtracted as non-specific binding. Unless otherwise noted, all analyses and coupling/capture steps were performed in HBS-P+ running buffer (10 mM HEPES, pH 7.4, 150 mM NaCl, 0.05 % Surfactant P20). Measurements were analyzed and fitted using the Biacore T200 Evaluation Software 3.0 (GE Healthcare). Data sets were exported to and further processed in GraphPad Prism 6.01 (GraphPad Software).

For biochemical and biophysical assays, peptide concentrations were reapproved using calibration-free concentration analysis (CFCA). Diffusion coefficients of peptides at 20 °C were calculated using the online Biacore tool termed Diffusion Coefficient Calculator [34] The molecular weight of each peptide was entered and all compounds were assumed to be in elongated shape (frictional ratio 2.5). The sample compartment temperature was set to 15 °C while the analysis was performed at 20 °C. Two variable dilutions were prepared and adjusted for each peptide in HBS-P+ buffer containing 1 mM ATP and 10 mM MgCl_2_. First, GST-PKA-Cα was captured to saturation with a flow rate of 30 μL/min resulting in capture levels of 750 RU to 2700 RU (high density). The adjusted peptide dilutions (ranging from as low as 1:150,000 to as high as 1:3,500,000) were subsequently injected for 45 s with flow rates of 5 μL/min and 100 μL/min, respectively, for each dilution. Between every peptide injection, PKA subunits were successfully regenerated by injecting elution buffer (described above) for 210 s with a flow rate of 30 μL/min.

To monitor association and dissociation of the biotin-labeled PKI^1–24^ analogs, interaction studies were adapted from [35]. Briefly, GST-PKA-Cα was injected to obtain capture levels of approximately 130–220 RU (low density) in each cycle. Two-fold peptide dilution series ranging from 50 pM to 28 nM were subsequently injected for 150 s (association) before initiating the dissociation phase by injecting buffer without analyte for another 150 s. Finally, regeneration of the immobilized α-GST antibodies was achieved by injecting 10 mM glycine (pH 1.9) for 30–60 s. All measurements were performed at 25 °C in HBS-P+ buffer containing 1 mM ATP and 10 mM MgCl_2_ at a flow rate of 30 μL/min. The equilibrium dissociation constant (K_D_) was subsequently calculated by dividing the dissociation rate constant (k_d_) from the association rate constant (k_a_) which were obtained by applying a 1:1 binding model (global fit, Langmuir conditions). Kinetic interaction analyses were performed in 3–5 independent repetitions with at least three protein preparations.

### 3.7. Microfluidic Electrophoretic Mobility Shift Assay (MMSA)

Kinase activity was determined by monitoring phosphorylation of the fluorescently-labeled substrate Kemptide (FITC-LRRASLG) in an MMSA to determine the amount of compound needed for half-maximal inhibition (IC_50_) as previously described [36]. Off-chip reaction mixtures were prepared in 384-well plates (Corning, low volume, nonbinding surface) containing 10 mM HEPES pH 7.4, 150 mM NaCl, 0.1 mg/ml BSA, 1 mM DTT, 0.1 % L-31, 1 mM ATP, 10 mM MgCl_2_, 250 μM Kemptide (GeneCust), 10 μM FITC-Kemptide (GL Biochem Ltd.) as well as 0.5 nM PKA-Cα or Cβ. Biotin-labeled peptides were tested over a 3-fold dilution series ranging from 17 pM to 9 μM. To remain maximum reaction velocity, Kemptide was applied at saturating concentrations of 260 μM [37]. After 45 min (PKA-Cα or 20 min (PKA-Cβ) of incubation in the dark at RT, samples were drawn into a four-sipper mode ProfilerPro Chip (Perkin Elmer) using a LabChip DeskTop Profiler (Caliper Life Sciences). While applying an upstream voltage of −1800 V and a downstream voltage of −150 V, substrate and product underwent electrophoretic separation in LabChip ProfilerPro Separation Buffer (PerkinElmer, Waltham, MA, USA) with a screening pressure of −1.7 PSI. Duplicate measurements were independently repeated 3–4 times with at least two separate protein preparations. Substrate conversion was plotted against log-scale inhibitor concentrations and fitted with nonlinear regression (sigmoidal dose-response curves) to determine IC_50_ values using GraphPad Prism 6.01 (GraphPad Software).

### 3.8. Cell Permeation Assays

HEK293 cells per well were seeded at 100,000 cells/well on 8-well chamber slides (BD Biosciences). Cells were grown overnight in DMEM medium with 10% fetal bovine serum. Next, 5 μM 5(6)-carboxyfluorescein-labeled peptides were added to the medium and incubated at 37 °C for 8 h before fixation in 2% paraformaldehyde. Slides were imaged using an Olympus IX71 microscope. Uptake experiments were repeated at least three times.

### 3.9. Cell-Based PKA Activity Assay

HEK293 cells were grown on 12-well culture plates (BD Biosciences). Cells were serum-starved for 24 h in serum-free DMEM with penicillin/streptomycin, glucose and L-glutamine. Peptides were added to cells at either 1, 5, or 10 μM concentrations for 6 h, followed by stimulation with 50 μM forskolin for 30 min. As a negative control, cells were treated with H89 (50 μM) for 30 min prior to forskolin stimulation. Cells were lysed in Laemmli sample buffer and analyzed by western blotting. Anti-phospho serine/threonine PKA substrate (1:1000, Cell Signaling Technology) or anti-tubulin (1:2000, DSHB) primary antibodies were used, followed by anti-rabbit IRDye 800CW (1:25,000) or anti-mouse IRDye 680LT secondary antibodies (1:30,000) (LI-COR Biosciences). Blots were imaged using an Odyssey Fc imaging system. Three independent replicates were performed.

## Figures and Tables

**Table 1:** IP20 Analogs developed as potential pseudosubstrate inhibitors for the catalytic subunit of Protein Kinase A (PKA-C) and summary of measured binding affinities (K_D_, [nM]) of peptides towards the human PKA catalytic isoforms Cα and Cβ1 as measured by fluorescence polarization (FP). Mean values of three independent measurements are given.

Compound	Sequence ^1^	Cα	Cβ1
**PKI^5–24^**	TTYADFIASGRTGRRNAIHD	n.d. ^2^	n.d. ^2^
**1**	βA-TTYADFIASGRTGRRNAIHD	0.7 ± 0.1	
**2**	βA-TTY*DFI*SGRTGRRNAIHD	5.2 ± 0.3	
**3**	PEG_3_-TTYADFIASGRTGRRNAIHD	0.8 ± 0.1	
**4**	PEG_3_-TTY*DFI*SGRTGRRNAIHD	5.4 ± 0.9	
**PKI^1–24^**	TDVETTYADFIASGRTGRRNAIHD	n.d. ^2^	
**5**	βA-TDVETTYADFIASGRTGRRNAIHD	0.4 ± 0.1	0.4 ± 0.1
**6**	βA-TDV*TTY*DFIASGRTGRRNAIHD	0.6 ± 0.1	0.8 ± 0.3
**7**	PEG_3_-TDVETTYADFIASGRTGRRNAIHD	0.5 ± 0.1	0.5 ± 0.1
**8**	PEG_3_-TDV*TTY*DFIASGRTGRRNAIHD	0.7 ± 0.2	0.9 ± 0.3

^1^ Stars represent positions where 2-(4′-pentenyl) alanine was inserted into the sequence. ^2^ n.d.–not determined.

**Table 2:** Summary of SPR analysis. Values were obtained from at least three independent measurements and are given with SD.

Compound	k_a_ (×10^6^ M^−1^ s^−1^)	k_d_ (×10^−3^ s^−1^)	K_D_ (nM)
**PKI^1–24^**	6.3 ± 1.7	7.2 ± 1.0	1.2 ± 0.2
**6**	8.0 ± 2.2	3.4 ± 0.4	0.5 ± 0.2
**8**	6.2 ± 1.4	3.5 ± 0.3	0.6 ± 0.2

**Table 3:** Summary of microfluidic electrophoretic mobility shift assays (MMSA) analysis. Values were obtained from at least three independent measurements and are reported with SD.

Compound	IC_50_ (nM)
**PKI^1–24^**	17.0 ± 3.4
**6**	24.8 ± 3.1
**8**	34.9 ± 5.2

## Supplementary Materials

The following are available online, Figure S1–S9; Table S1–S2.
