# Supplementary material for: A Stapled Peptide Mimic of the Pseudosubstrate Inhibitor PKI Inhibits Protein Kinase A
Source: Molecules. 2019 Apr 20;24(8):1567. doi: 10.3390/molecules24081567 (PMC6514771; doi:10.3390/molecules24081567)
Supplement: Supplementary file 1 [file molecules-24-01567-s001.pdf]

molecules

Supplementary Materials

A stapled peptide mimic of the pseudosubstrate inhibitor PKI inhibits Protein Kinase A

Jascha T. Manschwetus [1,#], George N. Bendzunas [2,#], Ameya J. Limaye [2], Matthias J. Knape [1,3], Friedrich W. Herberg [1,*] and Eileen J. Kennedy [2,*]

[1] Department of Biochemistry, Institute for Biology, University of Kassel, Heinrich-Plett-Str. 40, 34132 Kassel, Germany
[2] Department of Pharmaceutical and Biomedical Sciences, College of Pharmacy, University of Georgia, 240 W. Green St, 30602 Athens, GA United States
[3] Present address: Boehringer Ingelheim Pharma GmbH & Co. KG, Analytical Developments Biologicals, Birkendorfer Strasse 65, 88397 Biberach an der Riss, Germany.
# Authors contributed equally to this work
* Correspondence: ekennedy@uga.edu; Tel.: +1-706-542-6497 (E.J.K.); herberg@uni-kassel.de; Tel.: +49-561-804-4511(F.W.H.)

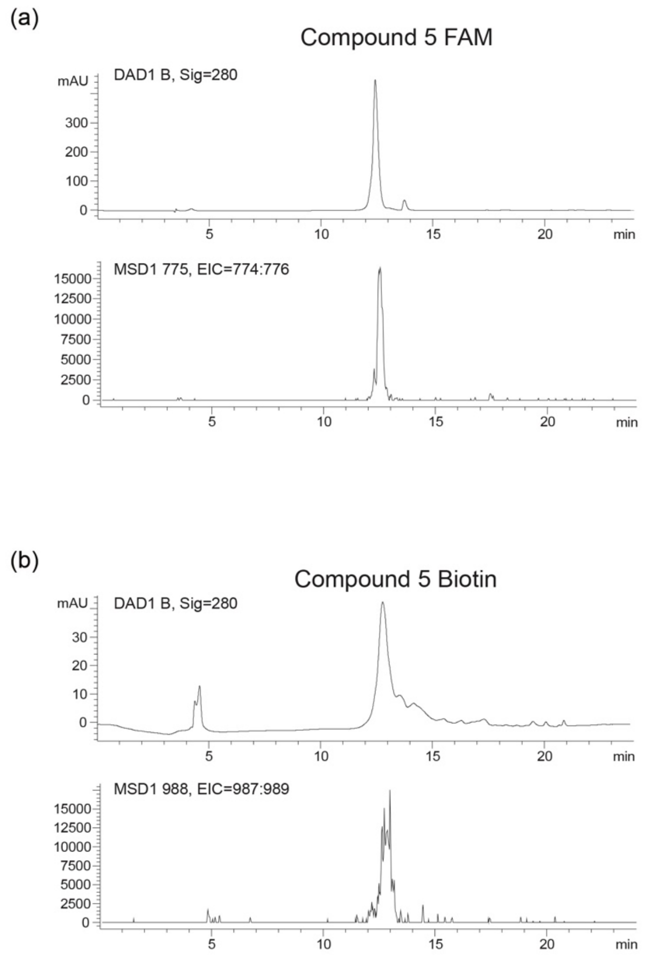

Figure S1. LC/MS spectra of HPLC-purified Compound 5. (**a**) Spectra of purified FAM-**5** is shown. The absorbance profile at 280 nm and extracted ion current (EIC) mass spectrometric detection (MSD) are included. The expected mass for (M+3)/3=775. (**b**) Spectra of purified Biotin-**5** is displayed. The expected mass for (M+2)/2=988.

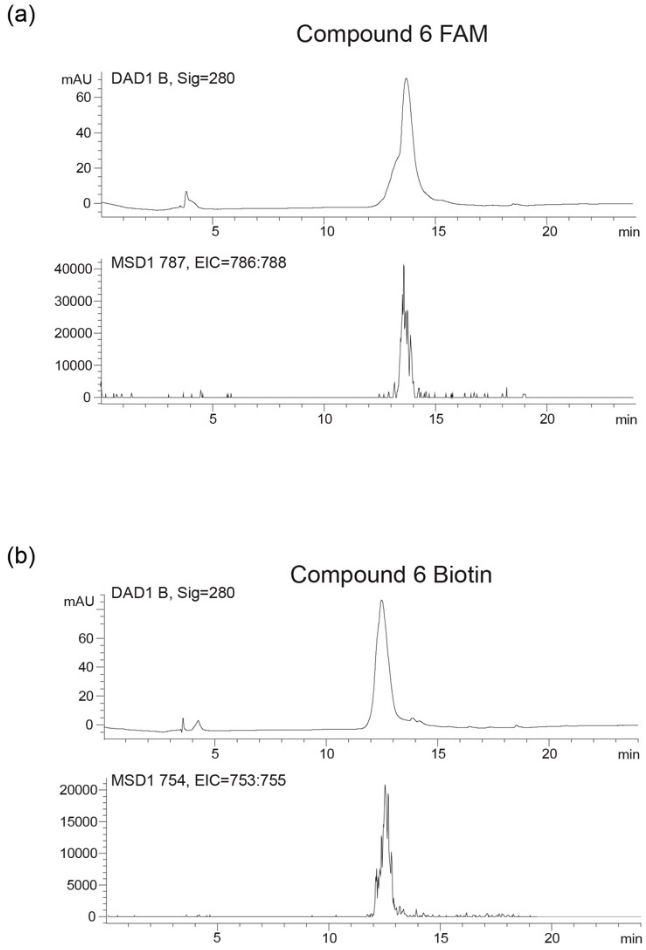

Figure S2. LC/MS spectra of HPLC-purified Compound 6. (**a**) Spectra of purified FAM-**6** is shown. The absorbance profile at 280 nm and extracted ion current (EIC) mass spectrometric detection (MSD) are included. The expected mass for (M+3)/3=787. (**b**) Spectra of purified Biotin-**6** is displayed. The expected mass for (M+3)3=754.

(a)

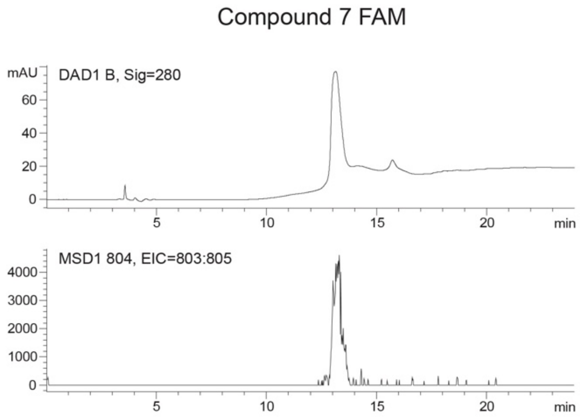

(b)

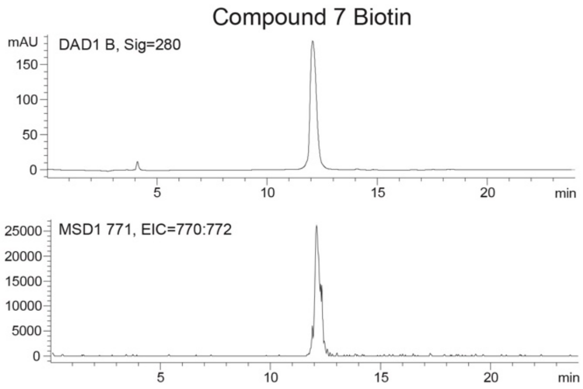

Figure S3. LC/MS spectra of HPLC-purified Compound 7. (**a**) Spectra of purified FAM-**7** is shown. The absorbance profile at 280 nm and extracted ion current (EIC) mass spectrometric detection (MSD) are included. The expected mass for (M+3)/3=804. (**b**) Spectra of purified Biotin-**7** is displayed. The expected mass for (M+3)/3=771.

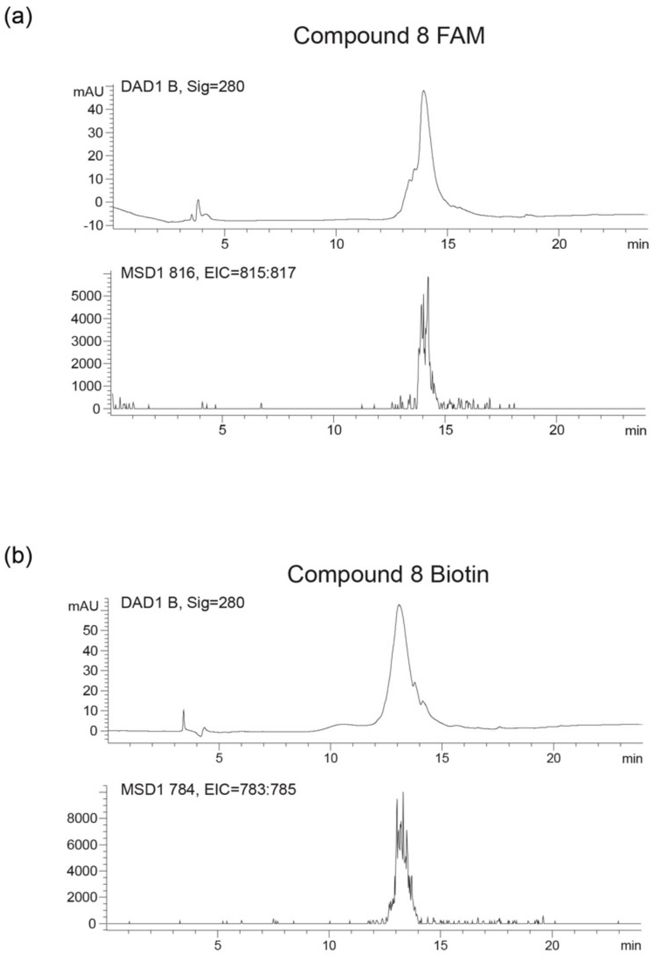

Figure S4. LC/MS spectra of HPLC-purified Compound 8. (**a**) Spectra of purified FAM-**8** is shown. The absorbance profile at 280 nm and extracted ion current (EIC) mass spectrometric detection (MSD) are included. The expected mass for (M+3)/3=816. (**b**) Spectra of purified Biotin-**8** is displayed. The expected mass for (M+3)/3=784.

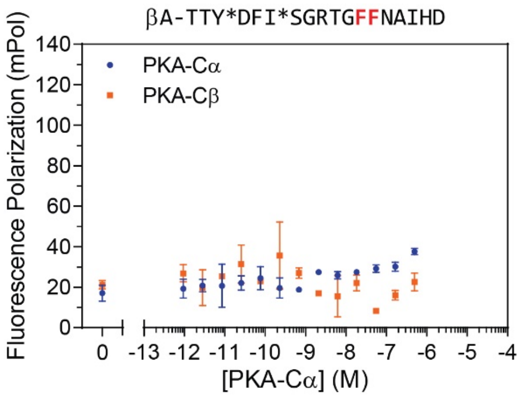

Figure S5. Direct binding measurements of a FAM-labeled negative control peptide using fluorescence polarization (FP): Binding measurements of a stapled PKI$^{5-24}$ analog (sequence on top) to PKA-Cα and PKA-Cβ demonstrate that the two basic Arg residues at P-2 and P-3 are critical for binding comparable to the wildtype protein PKI. This implicates that the interaction of this analog resembles the binding mode of unstapled PKI$^{5-24}$. No binding could be determined under given conditions in three independent measurements with three protein preparations for both PKA-C isoforms.

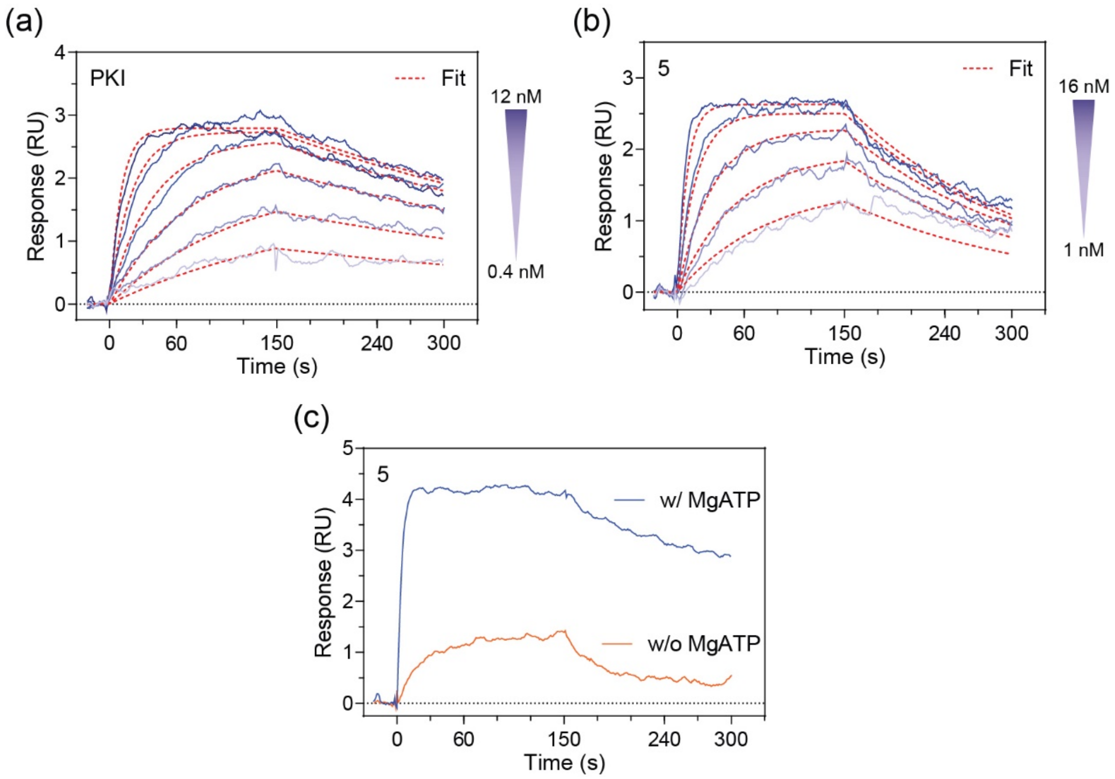

Figure S6. Kinetic interaction analyses by Surface Plasmon Resonance (SPR): (**a**) Binding measurements of the wildtype protein PKI$^{full\text{-}length}$ show subnanomolar affinities as a result of fast association and slow dissociation rate constants; (**b**) Binding measurements of Compound **5** reveal comparable binding to the stapled analog **6** (see main article); and (**c**) Binding of Compound **6** to PKA-C is highly dependent on the presence of 1 mM Mg^{2+} and 10 mM ATP as measured using running buffer with or without MgATP. Capture levels of GST-PKA-Cα were 127 and 125 RU for studies with and without MgATP, respectively.

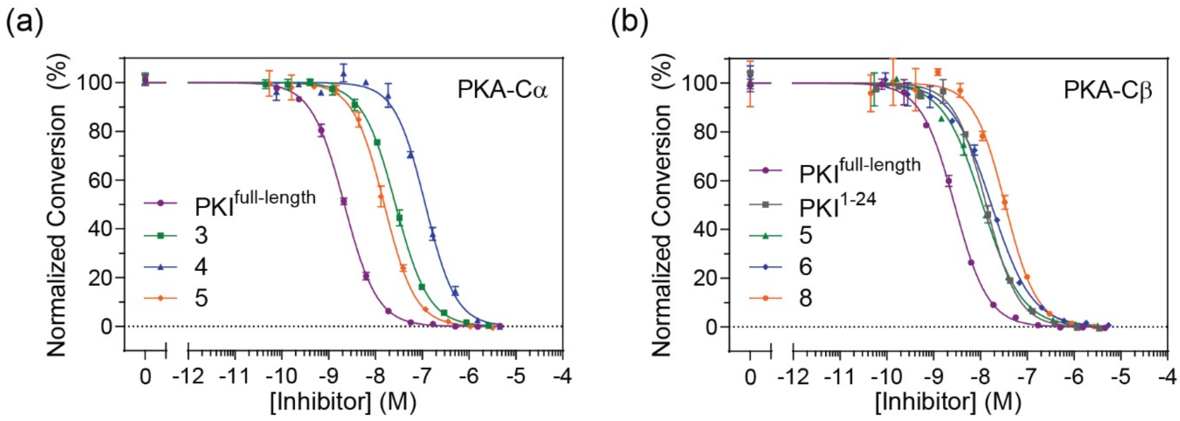

Figure S7. Inhibition of kinase activity was monitored using microfluidic electrophoretic mobility shift assays (MMSA): Phosphorylation of Kemptide by PKA-Cα (**a**) was monitored over a concentration range of inhibitor peptide **5** or PKI$^{\text{full-length}}$. The wildtype protein still shows increased inhibitory potency most likely due to additional interaction sites. (**b**) Inhibition of substrate peptide phosphorylation by PKA-Cβ1 was determined for PKI$^{\text{full-length}}$, PKI$^{1\text{-}24}$ and its derivatives **5**, **6** as well as **8**. The analyzed inhibitors were found to have IC$_{50}$ values that are comparable to PKA-Cα, thereby indicating that the hydrocarbon stapling of PKI$^{1\text{-}24}$ derived peptides does effect isoform specificity.

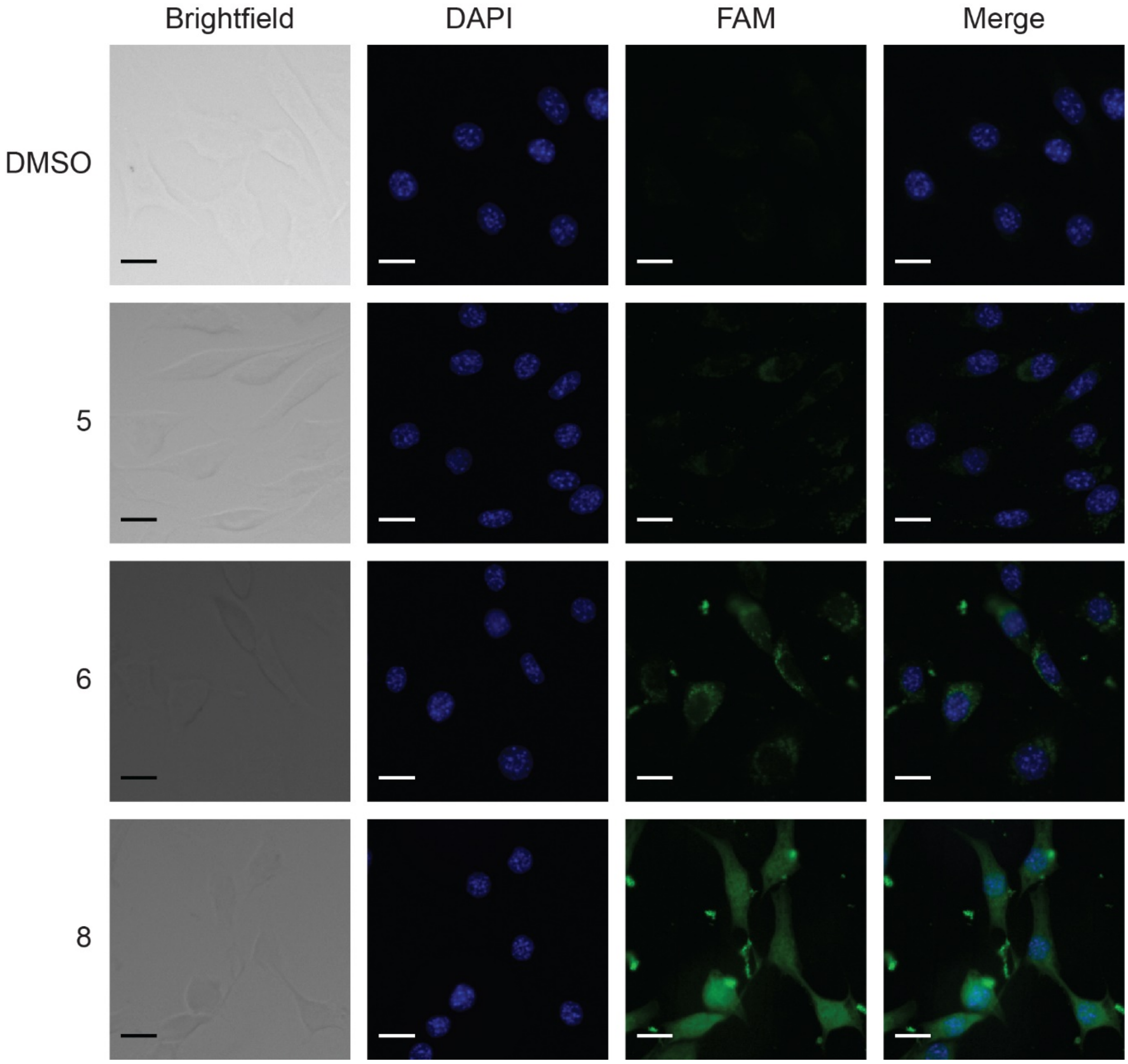

Figure S8. Cell permeation assay using NIH-3T3 cells: In DMEM containing 10 % fetal bovine serum, cells were grown to 50 % confluency in 8-chamber slides (CC2 treated, Lab-Tek II, Nalgene Nunc International) and treated with 5 µM of FAM-labeled peptides in DMEM for 4 (**6** and **8**) or 8 h (**5** and DMSO ctrl.), respectively. Subsequently, cells were washed, fixed in 2 % paraformaldehyde and embedded in mounting medium containing DAPI (ProLong Gold Antifade Mountant). Cells were imaged at 20 × magnification using a TCS SP5 microscope (Leica microsystems) before subtracting background (rolling ball radius: 100 µM) using ImageJ 1.51n. Compounds **6** and **8** were able to penetrate cells while the unstapled control peptide **5** could not permeate cells. Scale bars indicate 15 µm.

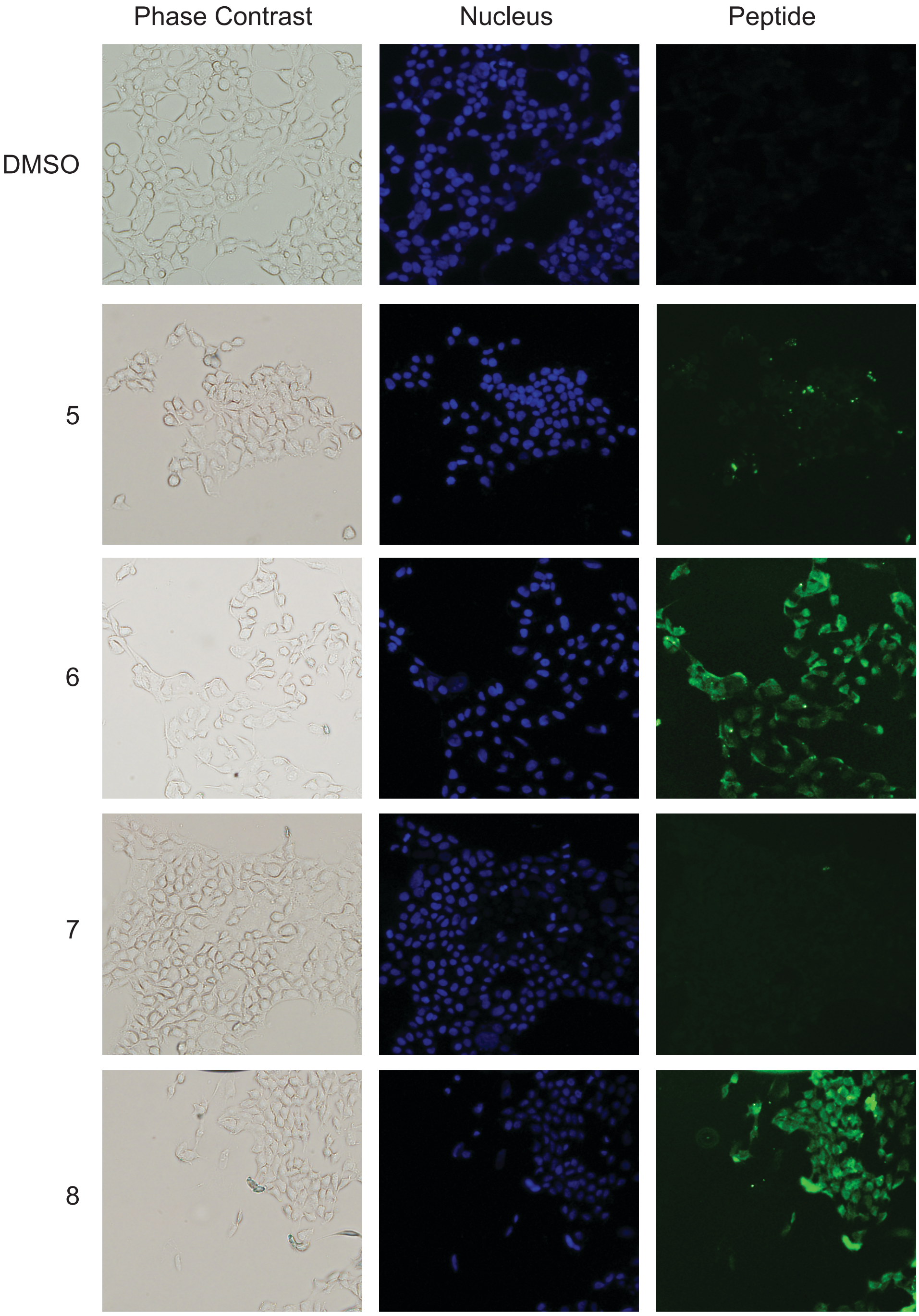

Figure S9. Un-zoomed view of cell uptake in HEK293 cells after 8 hr peptide treatment.

Table S1. Summary of SPR analysis. Values were obtained from one (PKI$^{full\text{-}length}$) or two (**5**) independent measurements and are given with SD. The kinetics of Compound **5** are similar to those of the stapled analog **6** (see main article).

Compound	k_a (× 10^6 M^{-1} s^{-1})	k_d (× 10^{-3} s^{-1})	K_D (nM)
**PKI$^{full\text{-}length}$**	8.3	2.4	0.3
**5**	6.2 ± 1.0	4.5 ± 2.2	0.7 ± 0.2

Table S2. Summary of additional IC_{50} values determined using MMSA analysis. Compound **3** shows half-maximal inhibition of PKA-Cα at a low nanomolar level while hydrocarbon stapling (**4**) decreased the inhibitory potency more than 3-fold. The IC_{50} values of both PKI$^{full\text{-}length}$ and PKI$^{1\text{-}24}$ as well as its derivatives (**5**, **6** and **7**) towards PKA-Cβ did not notably differ from those towards PKA-Cα. Values were obtained from two independent measurements with two protein preparations and are reported in nM with SD.

Compound	PKA-Cα	PKA-Cβ
**PKI$^{full\text{-}length}$**	2.2 ± 0.1[2]	2.5 ± 0.4
**3**	30.0 ± 4.4	n.d.
**4**	100 ± 23	n.d.
**PKI$^{1\text{-}24}$**	[1]	13.6 ± 0.4
**5**	15.8 ± 0.5	10.7 ± 0.7
**6**	[1]	19.1 ± 2.5
**8**	[1]	32.1 ± 2.6

[1] Values are shown in the main article; [2] n.d. – not determined.
